# Relationships among Sports Group Cohesion, Passion, and Mental Toughness in Chinese Team Sports Athletes

**DOI:** 10.3390/ijerph192215209

**Published:** 2022-11-17

**Authors:** Song Gu, Sheng Bi, Zhixun Guan, Xuemo Fang, Xulu Jiang

**Affiliations:** 1 College of Physical Education and Health Sciences, Zhejiang Normal University, Jinhua 321004, China; 2 College of Teacher Education, Zhejiang Normal University, Jinhua 321004, China

**Keywords:** cohesion, harmonious passion, obsessive passion, mental toughness

## Abstract

Background: Passion is an important motivational variable that profoundly affects athletes’ cognition, emotion, and behavior. This study constructed a mediating model to explore the mechanism of cohesion toward passion and mental toughness of Chinese team sports athletes and to investigate the mediating effect of harmonious passion and obsessive passion on cohesion and mental toughness. Methods: A questionnaire survey was conducted on 326 Chinese active athletes (M = 19.63, SD = 6.51) aged 14–26 years (54% male, 46% female) from eight sports. Results: Cohesion and its dimensions can positively predict athletes’ mental toughness, and ATG-T is more important in advantage analysis. The direct and indirect paths show that cohesion affects mental toughness through the mediating effect of harmonious passion and obsessive passion. Mediating effect model has a good fit and explained 22.1% of the variance in mental toughness. Conclusion: The relationship between cohesion, passion, and mental toughness reflects the psychological dynamic process from environment to motivation to sports performance. The development of team sports athletes’ mental toughness can be carried out from the following points. First, the team should define a sports goal and take the needs of members into account in goal-setting. Second, the sports team should build a team culture that is enterprising, inclusive, and cooperative and emphasizes members’ recognition of them. Third, the team should attach importance to the passion of the members and make good use of the team atmosphere. To protect the psychological health and long-term development of athletes, team culture should pay more attention to the cultivation of athletes’ harmonious passion. Improving cohesion is beneficial to athletes’ mental toughness in team sports. To protect the psychological health and long-term development of athletes, team culture should pay more attention to the cultivation of athletes’ harmonious passion.

## 1. Introduction

Mental toughness refers to the psychological qualities of an athlete to remain confident, focused, and motivated in a stressful situation [1]. Mental toughness is the most common term used to describe the mental ability of elite athletes in competitive sports [2,3]. Gucciardi believes that mental toughness can help athletes get rid of the impact of daily challenges, stressors, and major adversity so that they can continue to pursue their personal goals firmly and create high-level subjective and objective performance [4]. Because mental toughness has many positive effects on athletes, both scholars and coaches/athletes are very concerned about the formation mechanism of mental toughness. In the exploration of the antecedents of mental toughness, a series of qualitative theories have been put forward to explain the formation and development of mental toughness [3,4]. Their common view is that the formation of mental toughness will be affected by the interaction of environmental factors and toughness thinking [3,4,5,6,7]. As an important embodiment of the team environment, cohesion is considered the most important small group variable [8] which can affect athletes’ cognition, emotion, motivation, and other aspects [9]. Especially in team sports, cohesion plays a positive role in the process of group behavior and group effectiveness [9]. Among a series of potential influencing factors of mental toughness, passion, as a state of emotional engagement in individual participation activities [10], can deeply affect the cognitive regulation, emotional experience, and behavior pattern of athletes [11]. Moreover, passion can be stimulated by the external environment [10], and on this basis, a series of more positive adaptive results will be generated [12]. Some scholars even regarded passion as an important driving force for individual achievement goals and a necessary condition for achieving major achievements [11]. Therefore, clarifying the relationship among cohesion, sports passion, and mental toughness will help us to understand how the group environment affects athletes’ psychology and behavior to help improve team and individual competitive performance.

## 2. Theoretical Background and Hypotheses

Cohesion is a dynamic process reflected in the tendency of a group to stick together and remain united in pursuing its instrumental objectives and/or to satisfy members’ affective needs [8]. Cohesion consists of task and social cohesion, and it is usually regarded as an intermediate variable connecting sports teams and sports performance [9]. Task cohesion is the decisive force for a team to attract individuals and enable them to stay in the group. Then, social cohesion reflects interpersonal communication, such as personal feelings and emotional support among members. In the field of sports, the conceptual system proposed by Carron provides a guide for the study of sports cohesion [13]. Carron et al. (1981) improved the measurement method and proposed dimensions of cohesion through a model of sports group cohesion that includes group bonding and group attraction to individuals [9]. The four dimensions are individual attractions to group-task (ATG-T), individual attractions to the group social (ATG-S), group integration-task (GI-T), and group integration social (GI-S). This structure is widely used in the study of sports cohesion, in which the relationship between cohesion and sports performance has attracted more attention [1,13]. According to previous studies, sports events differences exist in the influence of cohesion on sports performance. Cohesion generally plays a role in promoting sports performance in collective events, and a few individual events (e.g., bowling and archery) are not closely related to cohesion [12].

In the field of elite sports, athletes often need to have multi-dimensional comprehensive abilities, such as physical strength, technology, tactics, and psychology, to successfully cope with the increasingly fierce competition situation [14]. Many of these athletes win at critical moments mostly because of their psychological advantages [2,3]. Mental toughness is the proper noun to describe these advantages [2]. At present, there are still some disputes about the component of mental toughness in the theoretical studies. The focus of these disputes is whether mental toughness is inherited or acquired. With the deepening of research, most scholars tend to define mental toughness as a type of state-like psychological resource, that is, mental toughness develops and changes with the change of situation and time. Based on the above views, the representative construct of mental toughness is the 3C model; it consists of confidence, constancy, and control. Gucciardi et al. (2009) found through qualitative research that these advantages will enable athletes to show more flexible coping strategies in life, training, and competition [15,16]. Mental toughness not only has stress-buffering effects on sports but also plays a protective role in favorable circumstances [4]. Mental toughness usually consists of self-confidence, constancy, and control [1]. At present, two main views exist on the formation of mental toughness. First, mental toughness originates from the personality hardiness of individuals and is affected mainly by individual factors [17]. Second, mental toughness will be affected by environmental factors [18]. For example, Zhang (1996) believes that athletes in the group environment can be affected by the emotional infection and behavior of other group members at any time [19]. Moreover, everyone will conform, obey, or depersonalize under the action of special normative factors of the group [19]. Based on this, the mental toughness of the team members will be more likely to be inspired and encouraged by cohesion.

At present, empirical research on the relationship between team cohesion and mental toughness is relatively rare. The reason may be that different sports events have different requirements for cohesion [12]. Therefore, implementing a large sample survey covering many events is difficult. After a qualitative analysis of the mental toughness characteristics of elite athletes in 31 sports, Fourie et al. (2001) pointed out that collective events athletes have a stronger mental toughness than individual events athletes, which is caused by the higher interdependence of group members. In collective events, athletes will be supported, guided, and motivated by their peers and coaches to better adapt to stress and recover quickly from adversity [5]. Connaughton et al. (2010) also highlighted the importance of the external environment, including motivational atmosphere, team development, and other aspects of team culture, in a strategic study of elite athletes developing mental toughness [18]. After studying a number of sports events, Ye (2014) suggested that individuals with higher social support and a high sense of achievement will contribute to the enhancement of mental toughness [19]. In sports, cohesion is an important source of social support for athletes [13]. In summary, considering cohesion as an attribute of team culture [20], we proposed the following hypothesis:

**Hypothesis** **1** **(H1).** *Cohesion and its constructs are positively related to mental toughness*.

In organizational behavior, passion is thought to be a powerful factor influencing individual preferences, importance, and motivations [21], and it will affect the internalized individual self-concept, behavior, or self-control [22]. Some scholars regarded passion as an important driving force for individual achievement goals and a necessary condition for realizing major achievements [23]. Hence, stimulating personal passion can affect their behavior. In the field of sports research, a few related topics show the potential role of passion in competitive performance [24]. Vallerand et al. (2008) believe that sports passion is a state of emotional engagement produced by individuals in the environment of participating in sports training or competition, which is characterized by strong, positive emotional motivation, internal driving force, and full emotional input [24]. Similar research by Swanson and Kent (2017) examined the role of passion in enhancing the performance of athletes and noted a strong correlation between these two constructs, especially during the last moments of the game when performance expectations are high [25]. Early passion studies paid more attention to the effect of passion on motivation [11] and did not emphasize the connection between passion and emotion. As research progressed, Vallerand (2010) proposed a dual model of harmonious and obsessive passion, which provides a new perspective for related research. Harmonious passion refers to promoting individuals to achieve their goals better and faster while effectively improving their mental health (e.g., life satisfaction, self-esteem, meaning in life, and vitality). Obsessive passion also pushes individuals to accomplish goals to some degree but breeds negative emotional experiences as well (e.g., burnout, anxiety, and oppression) [26,27]. Gucciardi (2009) studied cricketers and found that harmonious passion can negatively predict certain negative psychology and the negative emotions caused by an obsessive passion [14], including burnout and exhaustion, which are negatively correlated with mental toughness [28]. In addition, Lafreniere (2011) found that sports passion plays an important role in the self-control of behavioral outcome-driven processes [29]. Researchers of mental toughness also regard self-control as a basic component of mental toughness [1]. In summary, although different types of passion exist, they are closely related to athletes’ ability to emotional regulation and psychological pressure [11,30]. Therefore, passion may be related to mental toughness to some extent.

In addition, the dual model of passion provides speculation on the relationship between environment and passion [26]. It is believed that the internalization process of external motivation is an important mechanism for connecting environment and passion. On the basis of social cognitive theory [11], Zigarmi proposed a conceptual model of passion [10], and he suggested that individuals’ evaluation of their environment is the source of passion. Passion comes into being when evaluation brings lasting, positive, and meaningful happiness to individuals. Song (2015) pointed out that passion is a recent variable that can be used as a fusion of individual cognition, emotion, and motivation [31]. Notably, cohesion also plays an important role in athletes’ cognitive anxiety and motivation [13]. Reviewing the previous literature [14], we found that athletes with good mental toughness will conduct cognitive reappraisal through emotion regulation strategies. Hence, when individuals are challenged by physiology, technology, or situations, they can show the behavior of enduring pain and persevering in winning. Moreover, this case largely depends on the stimulation of the individual dynamic system of athletes.

Based on the above, this study believes that a functional relationship exists among cohesion, passion, and mental toughness, which reflects the dynamic psychological process from the environment to motivation to behavior choice. Exploring their relationship is likely to achieve results that go beyond the single study of cohesion and cognition and motivation and emotion and become a new direction in the field of cohesion research. Accordingly, this study proposes the following hypothesis:

**Hypothesis** **2** **(H2).** *Harmonious passion mediates the relationship between cohesion and mental toughness*.

**Hypothesis** **3** **(H3).** *Obsessive passion mediates the relationship between cohesion and athlete engagement*.

**Hypothesis** **4** **(H4).** *Harmonious passion and obsessive passion mediate the relationship between cohesion and mental toughness*.

## 3. Methods

### 3.1. Recruitment and Participants

Considering that n = 200 is the minimum sample size for SEM [32], a total of 445 provincial and national team players from Zhejiang, Heilongjiang, and Liaoning participated in the survey. All of them were team sports players, including football, basketball, cricket, volleyball, ice hockey, curling, and group aerobics. They have played sports for more than 3 years and have performed well in their respective sports. After deleting questionnaires with an overly short response time (less than 3 min) and those with answers that tend to be consistent, 326 were effectively recovered, with an overall effective rate of 73.3%.

### 3.2. Instruments

The questionnaire comprised three parts. First, we stated that this survey was being conducted voluntarily and anonymously. The answers to the questionnaire were available only to the researchers and not for commercial or any other use. Second, we collected the athletes’ basic information. Third, regarding the scale of the questionnaire used in this study, all questionnaire responses involved in this study were scored in five-point Likert scales, from strongly disagree (1) to strongly agree (5); the higher the score, the higher the recognition and acceptance of the item. The details are as follows:

#### 3.2.1. Group Environment Questionnaire (GEQ)

The GEQ was compiled by Carron (2010) and translated by Ma Hongyu [33]. This questionnaire was a special measurement questionnaire for sports cohesion, and CFA showed good reliability and validity when used in China: χ^2^/df = 2.69, RMSEA = 0.07, CFI = 0.95, GFI = 0.91, and NFI = 0.92. The questionnaire had 15 items, including four dimensions: ATG-T, ATG-S, GI-T, and GI-S, which represented the integration and involvement of task and social cohesion, respectively. The total amount and each dimension’s Cronbach’s α was 0.71~0.87.

#### 3.2.2. Sports Passion Questionnaire (SPQ)

The Sports Passion Scale was compiled by Vallerand Houlford (2003) and translated by the author of this study. In the use of Chinese athletes, good reliability and validity results were achieved. CFA showed: χ^2^/df = 2.01, RMSEA = 0.07, CFI = 0.92, GFI = 0.90, and NFI = 0.91. The scale had 16 items, including three dimensions: general passion, harmonious passion, and compulsive passion. The general passion scale is used mainly to identify whether the individual currently has passion, including four items. The Harmonious Passion Scale and the Forced Passion Scale were used to measure the degree of compulsive passion and harmonious passion. Each of them contained six items, and the total amount and each dimension’s Cronbach’s α was 0.74~0.81.

#### 3.2.3. Sports Mental Toughness Questionnaire (SMTQ)

The SMTQ was compiled by Sheard (2009) and translated by Wang Bin [34]. CFA showed high reliability and validity among Chinese professional athletes: χ^2^/df = 2.55, RMSEA = 0.06, CFI = 0.96, GFI = 0.90, and NFI = 0.93. The questionnaire consisted of 12 items, including three dimensions: confidence, constancy, and control. The total amount and each dimension’s Cronbach’s α was 0.74~0.82.

### 3.3. Data Collection

We provided questionnaires to the athletes after the training, and to ensure the quality of the study, athletes were informed of the purpose and confidentiality of the study. Then, they volunteered to answer a questionnaire that took approximately 10 min to complete and finally returned the questionnaire directly.

### 3.4. Validity and Reliability of the Instrument

Confirmatory factor analysis (CFA) was used to determine the internal validity of each structure. CFA showed the modified model fits the data well: CMIN/DF = 3.51, RMSEA = 0.09, GFI = 0.94, NFI = 0.93, and CFI = 0.95. Table 1 shows the internal consistency reliability and composite reliability. The Cronbach’s α values of each structure were greater than 0.7, indicating acceptable reliability [32]. In addition, the CR value of each structure was greater than 0.7, showing good comprehensive reliability [32]. AVE and FL values of each structure were higher than 0.4, indicating an acceptable level of convergence effectiveness (Table 1) [32].

### 3.5. Common Method Bias

CMB is a potential problem in the simultaneous collection of all structural data through self-reporting questionnaires. To reduce the interference of CMB on validity, this study applied a balanced item order, anonymous questionnaire measurement, and standardized measurement in the process of the questionnaire. Through Harman’s single factor test [32], we found that the variance explained by the first factor of principal component analysis was 30.4%, which was lower than the critical standard of 40%. CFA showed that the fit index of the 16-factor model (χ^2^ = 112.40, χ^2^/df = 3.51, RMSEA = 0.09, CFI = 0.95, GFI = 0.94, and NFI = 0.93) was significantly better than that of the single-factor model (χ^2^ = 2558.09, χ^2^/df = 2.85, RMSEA = 0.08, CFI = 0.77, GFI = 0.70, and NFI = 0.69).

### 3.6. Data Analysis

SPSS 22.0 (IBM, Armonk, NY, USA) was used to input the questionnaire data for descriptive analysis, reliability analysis, and hierarchical regression analysis. AMOS 21.0 (IBM, Chicago, IL, USA) was used for the CMB test, CFA, and mediating effect model analysis.

## 4. Results

### 4.1. Descriptive Analysis

From 1 June to 1 October 2021, a total of 445 questionnaires were distributed. After deleting the questionnaires with too short response time (less than 3 min) and consistent answers, 326 were effectively recovered. The effective rate was 73.3%. Among them, 175 cases were male (53.7%), and 151 cases were female (46.3%); 72 national second-level athletes (22.1%), 127 national first-level athletes (40.0%), 76 national elite athletes (23.3%), and 52 athletes lacked sports information. The average age of athletes was 19.63 years (SD = 6.51), and the average training time was 6.78 years (SD = 3.37). Detailed information is shown in Table 2.

### 4.2. Correlational Analysis

Table 3 shows the mean (M) and standard deviation (SD) of cohesion, sports passion, and mental toughness. Spearman analysis was used to test the correlation coefficient among cohesion, sports passion, and mental toughness. The results show that all variables were significant and correlated, with correlation coefficients between 0.35 and 0.66, supporting the validity of the overall data of the measurement model and the rationality of the topic packaging strategy.

### 4.3. Analysis of the Advantages of Different Dimensions of Cohesion in Predicting Mental Toughness

After controlling for gender, age, years of training, and sports performance, cohesion explained 18.4% of mental toughness (*β* = 0.31, *t* = 8.54, *p* < 0.001). Thus, cohesion has a significant positive effect on mental toughness. However, the effect of different dimensions of cohesion on mental toughness is unclear. To illustrate the explanatory effect of relevant dimensions, the advantage analysis method with model independence was used to calculate the change value of R^2^ after each explanatory variable was added to the sub-model without the variable itself to explain the relative contribution of each cohesive dimension to the athletes’ participation effect. The results (Table 4) show that all dimensions of cohesion were significantly positively correlated with mental toughness. H1 was verified; among them, ATG-T (37.23%) contributed the most to the explained variation.

### 4.4. Mediating Effect of Harmonious Passion and Obsessive Passion in Cohesion and Mental Toughness

**Figure 1:**
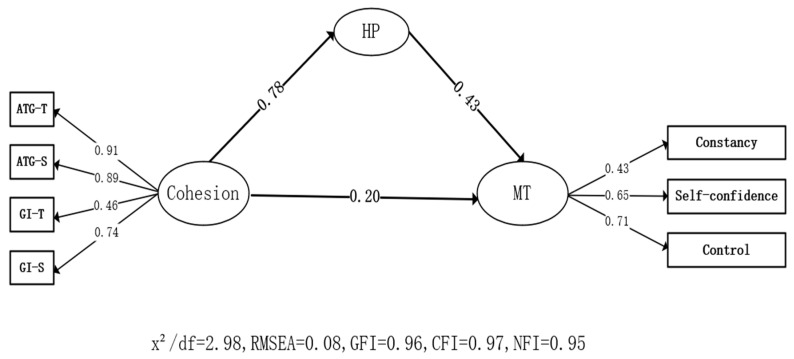
Mediating role of harmonious passion.

In the mediating effect model (Figure 1), cohesion significantly predicted harmonious passion (HP) (*β* = 0.78, *p* < 0.01), and harmonious passion significantly predicted mental toughness (MT) (*β* = 0.43, *p* < 0.01). This result indicates that an indirect effect existed in the path of cohesion and mental toughness, and the effect value was 0.78 × 0.43 = 0.34. H2 was verified.

**Figure 2:**
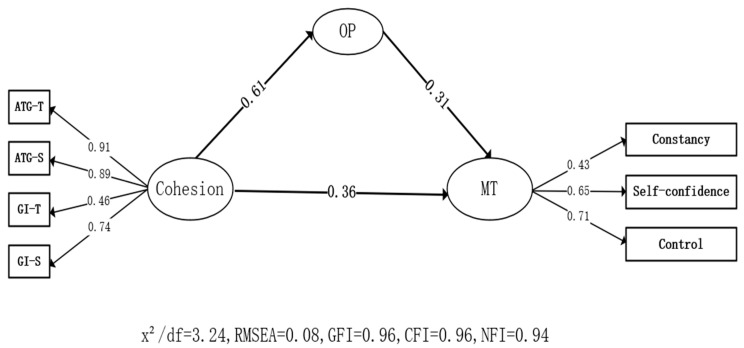
Mediating role of obsessive passion.

In the mediating effect model (Figure 2), cohesion significantly predicted obsessive passion (OP) (*β* = 0.61, *p* < 0.01) and obsessive passion significantly predicted athlete engagement (*β* = 0.3, *p* < 0.01), indicating that there was an indirect effect on the path of mental toughness in cohesion and athlete engagement, and the effect value was 0.45 × 0.39 = 0.18; thus, H3 was verified.

**Figure 3:**
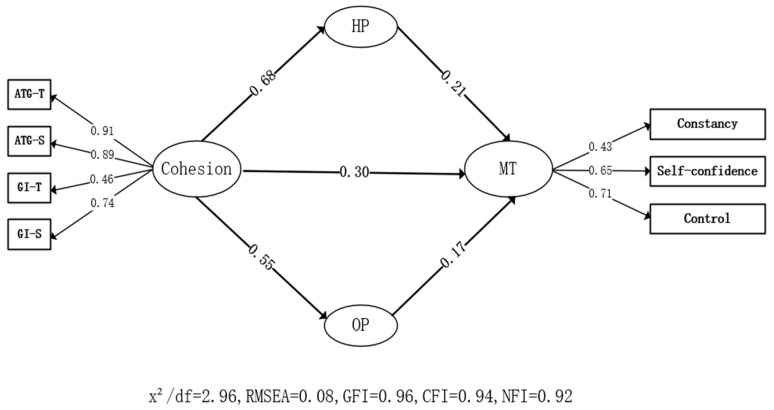
Mediating role of harmonious passion and obsessive passion.

In the mediating effect model (Figure 3 and Table 4), cohesion significantly predicted harmonious passion (β = 0.68, *p* < 0.01), obsessive passion (β = 0.55, *p* < 0.01), and mental toughness (β = 0.30, *p* < 0.01); harmonious passion–mental toughness pair (β = 0.21, *p* < 0.01) had a significant predictive effect; obsessive passion also had a significant predictive effect on mental toughness (β = 0.17, *p* < 0.01). These findings indicate that harmonious passion and obsessive passion had an indirect effect on the path of cohesion and mental toughness, and their influence values were 0.68 × 0.21 = 0.14, 0.55 × 0.17 = 0.09, respectively; therefore, H4 was verified. Among them, the total indirect effect of harmonious passion and obsessive passion accounted for 43% (Table 5). The R^2^ calculation of model fitting, cohesion, harmonious passion, and obsessive passion explained 22.1% of the change in mental toughness.

## 5. Discussion

### 5.1. Direct Effect of Cohesion on Mental Toughness

This study found that cohesion and its dimensions can positively predict mental toughness. The research results show that cohesion plays an important role in the formation of athletes’ mental toughness in collective sports events. Cohesion can not only increase athletes’ problem-solving strategies but also reduce athletes’ harmful evaluation of adversity [33]. In this way, athletes will have a strong sense of belonging and security, which will help them overcome setbacks and difficulties and achieve “rebound” in adversity. Through interviews, Chinese coaches usually use individual conversations and pre-match preparation meetings to improve morale, reduce stress, and ease or reduce team members’ anxiety. These methods are very effective. A good cohesive environment can establish a rich social connection for athletes. With the support of the team, athletes can obtain more resources to cope with stressful events as well as more positive coping methods, such as seeking help and solving problems [34].

Carron (2010) indicated that the development of sports cohesion includes three conditions. First, the group goal is clear and is recognized by members. Second, the needs, motives, and emotions among members are fully understood and supported. Third, those with prestige form the backbone that plays the role of regulating and communicating interpersonal relations, decision-making, organization, and leadership. When these conditions are established, the team can produce the energy amplification effect of mutual encouragement and improve behavior efficiency while achieving the self-defense effect of mutual protection from external pressure [35]. These gained effects provide an important guarantee for the growth and development of athletes, obtaining self-confidence, coping with adversity, and improving sports performance. Connaughton (2010) pointed out that the social support network and the team’s incentive atmosphere are conducive to the improvement of athletes’ mental toughness [18]. More support related to friendship and communication effect has a profound impact on athletes in improving sports performance [36,37,38], relieving psychological fatigue [13], rebuilding self-confidence [1,39] in the recovery period, coping with stress [15,16], and others. In addition, the advantage analysis showed that the four dimensions of cohesion could significantly positively predict mental toughness. Among them, ATG-T is a more important factor affecting athletes’ mental toughness. This case is similar to the previous results on the relationship between cohesion and athletic performance. According to the theory of management by objectives, a common goal is an important basis for bringing the whole team together. Song (2015) found that if the responsibility is assigned to every athlete, implementing “self-control” and “self-management” is more convenient, which can improve the athletes’ training enthusiasm and confidence and stimulate mental toughness to play a role in positive situations [31].

### 5.2. The Intermediary Role of Harmounious Passion and Obsessive Passion between Cohesion and Mental Toughness

In addition to the direct effect, this study shows that passion is not only the result of the internalization of external motivation but also an important internal factor in the process of cohesion affecting mental toughness. According to the self-determination theory [40], when people are in a social environment with high internal relevance needs, people naturally tend to assist and integrate external behavior regulation in the group. Vallerand’s definition of passion is rooted in this theory [40]. When members of a group are united and committed to achieving the same goals, it will promote the integration of environmental factors, improve the possibility of the group achieving these goals, and have an impact on the personal evaluation of the current organizational environment. Carron (2002) studied many factors related to cohesion, including personal factors, team factors, environmental factors, and leadership factors. In theory, the structure of passion is a cohesive association of personal factors because it is a form of motivation that can predict the persistence of sports groups [20]. According to the above point of view, the stimulation of sports harmonious passion should not only clarify the group task and the role of members but also deal with the interpersonal relationship of the team and conduct effective and positive emotional communication. This notion coincides with the “people-oriented management” of competitive sports talent training.

Cognitive interaction theory [31] believes that the generation of pressure is a dynamic and circular process, and the interaction between individuals and the environment is the basis for the formation of pressure. This process includes a series of factors, such as individual factors, environmental factors, cognitive evaluation, coping, and outcome. Previous studies showed that although players in a very united sports team will participate more persistently in training or competition, they still could often feel pressure and anxiety when they participate in training or competition [9]. On the basis of this, Fetcher et al. (2003) proposed a model of stress–emotion–performance based on the particularity of competitive sports [41]. This process emphasizes emotional orientation, that is, if individuals believe that they have the ability to control and cope with the emotional reactions they experience when encountering stress, then this emotion will help them to adopt a positive stress coping style to improve their competitive performance.

From the perspective of the dual model of passion, harmonious passion originates from the internalization process of motivation and emphasizes individual freedom. Harmonious passion often plays a positive role in individual achievements. Vallerand believes that the excellent performance of those passionate individuals is often partly driven by passion [11] because those passionate individuals can fully release themselves, pursue excellence, and overcome resistance [26]. Individuals with harmonious passion recognize and accept the value of sports, can control their emotions, and participate in training or competition freely, and they have the motivation to train or compete voluntarily. Schellenberg’s findings are similar [42], that is, athletes with harmonious passion will regard competition pressure as a challenge and adopt a method-oriented self-regulation process. At this time, the level of anxiety and burnout shown by athletes in the face of sports competitions will also be greatly reduced, such as coping with adversity, problem-centered coping, and task-oriented coping. Combining trait-coping theory [43], athletes with harmonious passion have the higher autonomous motivation and can better regulate negative and potential negative emotions. If they adopt correct self-regulation strategies to deal with stress in a pleasant and healthy psychological state, then they are more likely to show the characteristics of mental toughness. Amiot (2006) confirmed that athletes could eliminate negative thinking and stimulate positive emotions in a good group environment [44]. This case will significantly improve the athletes’ ability to cope with stress, reduce stress reactions, such as tension and anxiety, and then relieve emotional/physical exhaustion, causing them to stick to their efforts in adversity.

Notably, cohesion can positively predict mental toughness through obsessive passion. In response to this phenomenon, Jones (2008) believes that evaluation is an important factor in the generation of passion. Athletes will be forced to pursue the established sports goals, which shows an increase in compulsive passion. At this time, the individual’s internal actual sense of self-worth is insufficient [45], and their obsessive passion comes from the forced controlled internalization process of sports motivation [46]. Individuals with obsessive passion are forced to participate in training or competition instead of self-control due to internal or interpersonal pressure, uncontrollable sports excitement, etc. Therefore, it is often related to negative consequences, such as burnout [47], psychological pressure [25], and intention to retire [48]. However, in this case, obsessive passion did not hinder the formation of the mental toughness of Chinese athletes, which shows the complexity of mental toughness under the influence of the environment. Zhang (2009) explained, from the perspective of group dynamics, that athletes in a group environment could be affected by the emotions and behaviors of other members of the group at any time [33]. Moreover, everyone will follow the crowd and obey or depersonalize under the influence of group-specific normative factors [14]. The author holds that when the goals of individual athletes are inconsistent with the goals of the team, their obedience to team arrangements is greater due to organizational pressure. As time goes by, they begin to think about group task from a collective perspective. Emotional experiences such as fear and oppression will gradually fade away, followed by changes similar to the sublimation of personal interests to organizational interests. Thus, in the process, mental toughness is also improved.

Through further interviews, the study found that athletes with higher scores of obsessive passion are more eager to be recognized by coaches and peers. Considering that Chinese athletes often have a stronger collective consciousness and relationship needs, they seem more likely to project the target, tasks, and principles of group activities onto the standards of individual behavior. Although their behavior is compulsive, when they think that their efforts can achieve the group goals and gain the recognition of the group, they will also increase their expectations of achieving the group goals. The socially desirable response theory also puts forward a similar view [49]. When members believe that hard work will bring good performance evaluation and organizational rewards, and these rewards can meet the relationship needs of athletes, they have the motivation to make more efforts. However, when obsessive passion prevails, it may still produce more negative emotions, which may affect their mental health outside of sports. For this part of the athletes, sports teams should strive to create a good training environment, focusing on cultivating athletes’ harmonious passion for promoting the overall development of athletes.

### 5.3. Implications

In this study, cohesion was seen as an important factor in the organization and environment, which is an important source of social support for athletes. Passion is defined as a comprehensive concept integrating cognition, emotion, and behavior [11]. Mental toughness is the psychological trait of excellent athletes to take effective coping styles and maintain good performance under pressure. Mental toughness will be affected by cohesion and passion. This process can be explained as follows: in a very united sports team, the athletes themselves can deeply feel the warmth, encouragement, and trust of the team. In such an atmosphere, athletes can continually receive positive feedback in training and competition and enjoy sports experiences, to stimulate sports passion. This case will enable athletes to maintain sustained attention, persistence, and commitment to the goals set by the team.

Cohesion is a powerful engine for athletes to generate sports passion. The relationship between the two reflects the dynamic process in which group members unite to achieve their instrumental goals and produce a series of positive emotional experiences to meet the emotional needs of members. Considering the influence of obsessive passion and the greatest contribution of ATG-T on mental toughness, strengthening the emotional experience of athletes and clarifying the availability goal may be an effective means to mobilize cohesion resources in the short term to promote the mental toughness of athletes.

### 5.4. Limitations and Future Study

Although cross-sectional studies can provide valuable information, such studies cannot determine causality. This study does not consider possible adverse effects. Longitudinal tracking and experimental design may be used to test the findings of this study in the future. In the study of the mediation effect, only two dimensions of sports passion are selected, and deficiencies exist in the mechanism explanation. Future research can start from sports, team size, formal/informal groups, roles of leaders and members, team achievements, and even cultural background to improve this research’s findings. The results of this study are based on a sample of Chinese athletes, and the generalization of the research conclusions needs more research to verify.

## 6. Conclusions

The four hypotheses proposed in this study were supported. The total score of cohesion and all dimensions can significantly positively predict mental toughness, among which ATG-T played an important role in predicting mental toughness. Harmonious passion and obsessive passion played a partial mediating role between the cohesion and mental toughness of team athletes. In addition, the mediating effect accounted for 43% of the total effect. The mediating effect model constructed in this study has a good fit, which explains the mechanism of cohesion on mental toughness to a certain extent. The relationship among cohesion, passion, and mental toughness reflects the psychological dynamic process from the environment to motivation to behavior performance.

This study explored the role of cohesion in the formation of mental toughness in team sports. The development of team sports athletes’ mental toughness can be carried out from the following points. First, the team should define a sports goal and take the needs of members into account in goal-setting. Second, the sports team should build a team culture that is enterprising, inclusive, and cooperative, and emphasizes members’ recognition of them. Third, the team should attach importance to the passion of the members and make good use of the team atmosphere. To protect the psychological health and long-term development of athletes, team culture should pay more attention to the cultivation of athletes’ harmonious passion.

## Figures and Tables

**Table 1:** Reliability and validity analysis.

Variables	Variables	Items	Cronbach’s α	CR	AVE	FL
Cohesion	ATG-T	3	0.77	0.79	0.56	0.72–0.80
	ATG-S	4	0.83	0.84	0.56	0.71–0.79
	GI-T	4	0.77	0.71	0.51	0.49–0.81
	GI-S	3	0.71	0.70	0.44	0.40–0.50
Passion	General Passion	4	0.89	0.89	0.66	0.79–0.83
	Harmonious Passion	6	0.90	0.91	0.62	0.68–0.84
	Obsessive Passion	6	0.88	0.88	0.55	0.63–0.82
Mental Toughness	Self-confidence	4	0.73	0.70	0.50	0.43–0.66
	Constancy	4	0.75	0.77	0.54	0.60–0.67
	Control	4	0.71	0.74	0.51	0.65–0.98

**Table 2:** Descriptive statistics of selected groups.

Variables	N	%
Total	326	100
Gender		
Male	175	53.7
Female	151	46.3
Sports level		
National elite athletes	76	23.3
National first-level athletes	127	39
National second-level athletes	72	22.1
Lack sports information	51	15.6
Games		
Football	63	19.3
Basketball	74	22.7
Cricket	47	14.4
Volleyball	36	11.1
Ice hockey	20	6.1
Curling	11	3.4
Group aerobics	47	14.4
Others	28	8.6

**Table 3:** Descriptive statistics of model variables and correlations among model variables.

Component	M	SD	1	2	3	4
Cohesion	4.09	0.53	--			
Harmonious Passion	4.15	0.64	0.66 ***	--		
Obsessive Passion	3.74	0.74	0.51 ***	0.62 ***	--	
Mental Toughness	3.40	0.38	0.43 ***	0.41 ***	0.35 ***	--

Note: *** *p* < 0.001.

**Table 4:** Cohesion constructs predicting relative contribution of mental toughness.

Multidimensional Dimension of Cohesion	R^2^	Value-Added Contribution			
		X_1_	X_2_	X_3_	X_4_
—	—	0.167	0.136	0.071	0.126
X_1_	0.167	—	0.003	0.013	0.013
X_2_	0.136	0.034	—	0.020	0.025
X_3_	0.071	0.108	0.085	—	0.071
X_4_	0.126	0.054	0.034	0.016	—
X_1_X_2_	0.170	—	—	0.012	0.011
X_1_X_3_	0.180	—	0.002	—	0.009
X_1_X_4_	0.172	—	0.001	0.008	—
X_2_X_3_	0.156	0.026	—	—	0.015
X_2_X_4_	0.161	0.021	—	0.011	—
X_3_X_4_	0.143	0.046	0.029	—	—
X_1_X_2_X_3_	0.182	—	—	—	0.007
X_1_X_2_X_4_	0.182	—	—	0.008	—
X_1_X_3_X_4_	0.188	—	0.001	—	—
X_2_X_3_X_4_	0.172	0.018	—	—	—
X_1_X_2_X_3_X_4_	0.189	—	—	—	—
Relative importance analysis	—	0.070	0.047	0.026	0.045
Predicted variance percentage	—	37.23	25.00	13.83	23.94

Note: X_1_, X_2_, X_3_, and X_4_ indicate ATG-T, ATG-S, GI-T, and GI-S, respectively.

**Table 5:** Effect analysis of latent variables.

Influence Path	Standardized Effect Value	Significance	%
Cohesion→Mental toughness	0.30	***	56.6
Cohesion→Harmonious passion→Mental toughness	0.68 × 0.21 = 0.14	***	26.4
Cohesion→Obsessive passion→Mental toughness	0.55 × 0.17 = 0.09	***	17.0
The total indirect effect	0.14 + 0.09 = 0.23	***	43.4
The total effect	0.30 + 0.23 = 0.53	***	——

Note: *** *p* < 0.001.

## Data Availability

The data in the study are not publicly available in order to protect privacy of the participants.
